# Susceptibility of ten rice brands to weevil, *Sitophilus oryzae* L. (Coleoptera: Curculionidae), and their influence on the insect and infestation rate

**DOI:** 10.1186/s42269-020-00459-w

**Published:** 2021-01-06

**Authors:** Chilee Okpile, Usman Zakka, Luke Chinaru Nwosu

**Affiliations:** Department of Crop and Soil Science, Faculty of Agriculture, University of Port Harcourt, Rivers State, P.M.B. 5323, Port Harcourt, Nigeria

**Keywords:** Branded, F_1_ progeny emergence, Resistance, Rice security, Royale stallion

## Abstract

**Background:**

Susceptibility studies keep farmers, managers and household users informed and enhance breeding program’s testing against infestation and damage by storage insect pests. Therefore, laboratory tests were carried out to examine the susceptibility of ten rice brands to rice weevil, *Sitophilus oryzae* L. (Coleoptera: Curculionidae), infestation under temperature and relative humidity of 25 ± 2 °C and 75 ± 5%, respectively. The specific objectives of the study were to identify some commercially available rice brands with resistance to *S*. *oryzae*, by determining whether brand difference influences insect body weight at emergence and whether infestation is related to brand palatability and appearance. The ten brands used for the study were royale stallion, Mama royale, parboiled rice, Mama gold, white rice, Super eagle, Indian rice, champion rice, Abakiliki rice and Mama Africa, and standard methods were used to achieve the objectives. The indices measured were F_1_ progeny emergence, grain weight loss and frass accumulation.

**Results:**

The results showed that Abakiliki rice was poor in both palatability and appearance, whereas Super eagle was the most palatable and white rice was visually excellent. The results further showed that no brand was resistant to infestation and damage by *S*. *oryzae*. Males that were bred on the different rice brands did not differ in weight at emergence, but the weight of females at emergence was influenced by brand difference, and this suggests that female insects are more sensitive to brand difference at weight gain.

**Conclusion:**

Palatability and appearance were important in the susceptibility of rice brands to *S*. *oryzae*. The proliferation of diverse genotypes of rice (branded) with enhanced resistance to *S*. *oryzae* should be strongly encouraged to reduce susceptibility and increase rice security.

## Background

Rice, *Oryzae sativa* L. (Poaceae), is one of the most widely grown cereals with high global recognition and food value (January et al. 2020). Rice grain serves as staple for more than 60% of the world’s population, and it nourishes the human body with protein, carbohydrate, fiber, minerals and vitamins (Ashamo 2005; Akhtar et al. 2015; Da Silva Costa et al. 2016; Babendreier et al. 2020). Rice is an elite diet in many parts of the world, and it is held at very high esteem, especially during festivals. Both adults and children love rice, and its amino acid content and caloric value are reasonable. Ashamo (2005) has further highlighted the importance of rice. Briefly, rice flour and honey are used to make delicious bread. Rice can be processed for starch, wine, beer and spirits. The rice bran serves as livestock feed, while oil from bran is used for cooking and soap production. The importance of rice is high and should not be over-emphasized. The whole activities on rice, starting from cultivation, processing, manufacture of other products from rice and delivery provide employment opportunities. Rice grains are stored for several important reasons for food security, planting and trading to make financial gains. In times of emergencies such as corona virus disease outbreak and lockdown, most families would also store rice for prolonged consumption. Unfortunately, in rice grain storage, insect pest attack occurs (Khan and Halder 2012). The insect pests which attack rice in stores include *Sitophilus oryzae* L., *Sitotroga cerealella* (Olivier), *Rhyzopertha dominica* (Fabricius), *Trogoderma granarium* (Everts) and *Tribolium castaneum* (Herbst), and among these insects, the rice weevil, *Sitophilus oryzae* L. (Coleoptera: Curculionidae), is the most important species (Akhtar et al 2015). This is because the weevil can feed directly on intact grain kernels of rice which serve as natural host to the pest (Batta 2004). The activities of this primary pest culminate in loss of grain weight, nutrients and seed viability, and their activities make grains vulnerable to contamination by mites and fungi (Da Silva Costa et al. 2016; Zakladnoy 2018). The occurrence of the weevil in rice for milling and bread reduces the commercial value, and therefore, the insect should not even be seen in rice brands for it to be highly marketable.

Different strategies are available for the control of *S*. *oryzae* infestation in storage. Phosphine insecticide has been very effective; however, problems of resistance limit its sustainability, and when this occurs, alternative measures are sought by rice growers (Lee et al. 2001; Hossain et al. 2014). In both local and international markets, various rice brands are marketed and the competition is high. A rice brand that will attract high demand will have good appearance and should be palatable. This is in line with the demands of nature because human beings love good-looking and palatable commodities. Incidentally, the roles of appearance and palatability in the susceptibility of commercially available rice brands to *S*. *oryzae* have been grossly understudied. This is one feature the present study hopes to tackle. The objectivities of this study are to (1) identify commercially available rice brands with resistance to *S*. *oryzae*, (2) determine whether brand difference influences insect body weight at emergence and (3) determine whether infestation rate is related to brand palatability and appearance.

## Methods

### Experimental site

The experiment was conducted in the Crop Protection Laboratory of the Department of Crop and Soil Science, Faculty of Agriculture, University of Port Harcourt, Nigeria. The temperature and relative humidity of the experimental site were 25 ± 2 °C and 75 ± 5%, respectively, during the period of the study. The site had sufficient oxygen penetration (windows were left open) for the optimum performance of the rice weevil, *Sitophilus oryzae* L. insect.

### Rice brands

Ten different brands of rice which are commercially available were used for the study. These were royale stallion, Mama royale, parboiled rice, Mama gold, white rice, Super eagle, Indian rice, champion rice, Abakiliki rice and Mama Africa. They were purchased at random from rice stores in Port Harcourt city, Rivers State, Nigeria. Four kilograms of each sample was put in a small black polythene bag and carefully tied and labeled with the aid of a string. The samples were transported to the laboratory for palatability and appearance tests which were carried out immediately.

### Palatability and appearance tests

Two kilograms of each of the ten rice brands was cooked separately with water only, for 30 min on a Binatone gas cooker until soft to determine palatability. To rate the brands according to palatability, 50 panelists (drawn from University of Port Harcourt, Nigeria) were randomly selected and served with each of the cooked rice brands coded with a letter to reduce prejudice. Each panelist ate a spoon full of each rice brand and rinsed mouth with water before tasting the other brand to prevent the cross-palatability effect. The panelists rated the palatability of the rice brands using a scale of 1 to 5 scores in a form provided. The scores of 1 to 5 corresponded to poor, fair, fairly good, good and excellent. Twenty grams of each rice brand (also coded) was presented alongside cooked samples for visual examination by the panelists who also scored their observations as described above.

### Insect culture

Local rice infested by rice weevil (*S*. *oryzae*) was obtained from the open market in Choba, Rivers State, Nigeria, and kept in five separate 1-L Kilner jars in the laboratory where weevil identity was confirmed by an expert. The rice weevils were sieved out using a plastic laboratory test sieve and transferred to five separate 1-L Kilner jars containing fresh Abakiliki rice. After 7 days, when the weevils have fed and oviposited, they were sieved out. The setup was allowed to stay for 35 days until emergence of new progenies began. New adult progenies of similar age (7 days old) were used for the experiment.

### Susceptibility test

For susceptibility test, only fresh intact rice grains were used. Damaged grains and other irrelevant materials were sorted out and discarded. Thirty grams of all rice brands weighed using the sensitive electronic Mettler balance (model mp 2003) was disinfested with cold temperature by keeping them in a refrigerator for 7 days, and the grains were considered completely disinfested (Khan and Halder 2012). The disinfested grains were spread on a muslin cloth and allowed to acclimatize at room temperature and relative humidity for 72 h to bring them to normal condition. The average moisture content of the rice samples (11.5%) was determined using the standard oven method (Khan and Halder 2012). Twenty grams of standardized grains of each rice brand was introduced separately into a 100-ml capacity jar. Two pairs (2♀ + 2♂) of 7-day-old *S*. *oryzae* sexed morphologically as described by Halstead (1963) were released into each jar and covered with muslin net, arranged in a completely randomized design on the workbench and replicated four times. The parent weevils were left for 7 days to feed and lay eggs, after which they (living and dead) were sieved out. The containers were kept undisturbed under the condition of normal room temperature and relative humidity (25 ± 2 °C and 75 ± 5%). Insect count per brand began 35 days post-infestation when the F_1_ progenies have started emerging up to 56 days post-infestation duration. The weight (g) of F_1_ male and female progenies of *S*. *oryzae* was also recorded per rice brand. The seeds in each container were measured to determine percent weight loss on the 57th day post-infestation. The weight of frass (g) was also measured at the same day.

### Statistical analysis

Data of number of emerged progenies, weight of emerged progenies and weight of frass were analyzed using one-way analysis of variance, and significantly different means were separated using Tukey’s studentized range (HSD) at *α* = 5%. The software was Statistical Package for the Social Science (SPSS), version 19.0. Palatability and appearance were scored, described and presented in a table, while associations of rice brand palatability and appearance with susceptibility indices were assessed using electronic scatter plots.

## Results

### Palatability and appearance of rice brands

Table 1 shows the palatability of ten rice brands screened for susceptibility to *S*. *oryzae*. Their palatability ranged from poor to excellent. Abakiliki brand (a local rice variety) was the poorest in taste, while Super eagle was the most palatable rice. Mama gold and Mama Africa as well as Champion rice were equally rated fairly good and good. Table 2 presents the appearance of the rice brands. Abakiliki rice appeared poor, while white rice was visually excellent. Mama gold and Mama Africa appeared good, while the rest were fairly good except Royale stallion that was fair in appearance.

**Table 1:** Palatability of ten rice brands ascertained by panelist of rice consumers

Serial number	Rice brands	Mean scores	Mean observation^a^	Modal observation
1	Royale stallion	2.70	Fairly good	Fair
2	Mama royale	3.10	Fairly good	Fairly good
3	Parboiled rice	2.70	Fairly good	Fair
4	Mama gold	3.50	Good	Fairly good and good
5	White rice	2.70	Fairly good	Fair
6	Super eagle	4.30	Good	Excellent
7	Indian rice	2.70	Fairly good	Fair
8	Champion rice	3.80	Good	Good
9	Abakiliki	1.10	Poor	Poor
10	Mama Africa	3.80	Good	Fairly good and good

Scores 1 = poor, 2 = fair, 3 = fairly good, 4 = good and 5 = excellent

^a^Representative observation was based on approximated (nearest whole number) mean scores

**Table 2:** Appearance of ten rice brands ascertained by panelist of rice consumers

Serial number	Rice brands	Mean scores	Mean observation^a^	Modal observation
1	Royale stallion	2.40	Fair	Fair
2	Mama royale	3.00	Fairly good	Fairly good
3	Parboiled rice	3.10	Fairly good	Fairly good
4	Mama gold	3.70	Good	Good
5	White rice	4.70	Excellent	Excellent
6	Super eagle	3.30	Fairly good	Fairly good
7	Indian rice	2.90	Fairly good	Fairly good
8	Champion rice	3.00	Fairly good	Fairly good
9	Abakiliki	1.10	Poor	Poor
10	Mama Africa	4.00	Good	Good

Scores 1 = poor, 2 = fair, 3 = fairly good, 4 = good and 5 = excellent

Representative observation was based on approximated (nearest whole number) mean scores

### Susceptibility of rice brands to infestation by *Sitophilus oryzae*

Table 3 presents the number of new adults which emerged from the different brands of rice, grain weight loss caused to rice brands by *S*. *oryzae*, and the weight of frass generated in the different rice brands. The result shows that significantly higher number of new adults emerged from Mama royale and Super eagle rice brands, though it did not differ statistically from those recorded on Royale stallion, White rice, Champion rice, Abakiliki and Mama Africa. Least number of new progenies emerged from Indian rice; however, it did not differ statistically from Parboiled rice and Mama gold. White rice experienced significantly higher weight loss, and this did not differ from weight loss suffered by other brands except Super eagle which significantly had lower weight loss. Indian rice had the lowest weight loss, and this was a significant observation. Significant differences occurred in the quantity of frass generated in the different rice brands. Frass was not detected in Royale stallion rice, Parboiled rice and White rice. Super eagle had the highest frass accumulation, and this was followed by Abakiliki rice.

**Table 3:** Number of new F_1_ progenies, grain weight loss and frass weight generated by *Sitophilus oryzae* in ten rice brands

Serial number	Rice brands	Number of emerged F_1_ progenies^a^	Weight loss (%)	Weight of frass (g)
1	Royale stallion	11.00 ± 1.22ab	20.08 ± 0.0750ab	0.00 ± 0.0000b
2	Mama royale	18.25 ± 3.79a	19.73 ± 0.1665abc	0.21 ± 0.1091ab
3	Parboiled rice	6.75 ± 1.25b	20.08 ± 0.0862ab	0.00 ± 0.0000b
4	Mama gold	9.50 ± 1.66ab	20.36 ± 0.1285ab	0.19 ± 0.0211ab
5	White rice	14.75 ± 1.25ab	20.53 ± 0.9844a	0.00 ± 0.0000b
6	Super eagle	18.75 ± 6.34a	18.86 ± 0.3037c	0.41 ± 0.2807a
7	Indian rice	5.50 ± 1.19b	16.68 ± 0.0377d	0.01 ± 0.0004b
8	Champion rice	14.25 ± 5.57ab	19.38 ± 0.2266bc	0.14 ± 0.0438ab
9	Abakiliki	15.00 ± 2.68ab	19.55 ± 0.1190abc	0.16 ± 0.0401ab
10	Mama Africa	13.00 ± 2.94ab	19.45 ± 0.2594bc	0.15 ± 0.0581ab
	*F* statistic	*F*_9,30_ = 1.81	*F*_9,30_ = 9.680	*F*_9,30_ = 1.73
	*P* value	0.11	< 0.0001	0.125

Data are means ± SEM of four replications

Mean values in a column with same letter are not significantly different by HSD (*α* = 0.05)

### Weight of male and female F_1_ progenies of *Sitophilus oryzae*: effect of brand difference on insect body weight and associations of palatability and appearance with insect body weight

The mean weights of male and female *S*. *oryzae* that emerged from the ten rice brands are presented in Table 4. There was no significant difference among male weevils that emerged from the different rice brands. There were significant differences in the weight of female *S*. *oryzae* that emerged from the rice brands. Mama royale produced female weevil of highest weight, though this did not differ significantly with weight of weevils which bred on Mama gold, White rice, Super eagle, Indian rice and Abakiliki. The least female *S*. *oryzae* weight was recorded on Champion rice and Mama Africa which did not differ from female weight recorded on Royale stallion. Brand difference did not vary statistically with the weight of male F_1_ progenies of *S*. *oryzae*. However, brand difference caused variation among the weights of female F_1_ progenies. Palatability of rice did not have positive influence on the weight of male and female F_1_ progenies of *S*. *oryzae* (*Y* = − 699.0*X* + 4.696 and *Y* = − 271.1 + 3.826, respectively). Good appearance of rice had a negative effect on the weight of male and female F_1_ progenies too (*Y* = − 707.1*X* + 4.796 and *Y* = − 220.3 + 3.759, respectively).

**Table 4:** Mean weight of male and female *Sitophilus oryzae* L. emerged from ten rice brands

Serial number	Rice brands	Mean weight of males (g)	Mean weight of females (g)
1	Royale stallion	0.0022 ± 0.0003a	0.0026 ± 0.0003bc
2	Mama royale	0.0027 ± 0.0003a	0.0038 ± 0.0006a
3	Parboiled rice	0.0020 ± 0.0002a	0.0029 ± 0.0004abc
4	Mama gold	0.0025 ± 0.0003a	0.0030 ± 0.0003ab
5	White rice	0.0023 ± 0.0004a	0.0030 ± 0.0004ab
6	Super eagle	0.0024 ± 0.0003a	0.0036 ± 0.0005ab
7	Indian rice	0.0029 ± 0.0004a	0.0032 ± 0.0002ab
8	Champion rice	0.0021 ± 0.0002a	0.0019 ± 0.0002c
9	Abakiliki	0.0025 ± 0.0006a	0.0031 ± 0.0003ab
10	Mama Africa	0.0021 ± 0.0002a	0.0019 ± 0.0003c
	*F* statistic	*F*_9,30_ = 0.730	*F*_9,30_ = 3.010
	*P* value	0.683	0.004

Data are means ± SEM of four replications

Mean values in a column with same letter are not significantly different by HSD (*α* = 0.05)

### Infestation rate of rice grains and its associations with brand palatability and appearance

**Fig. 1:**
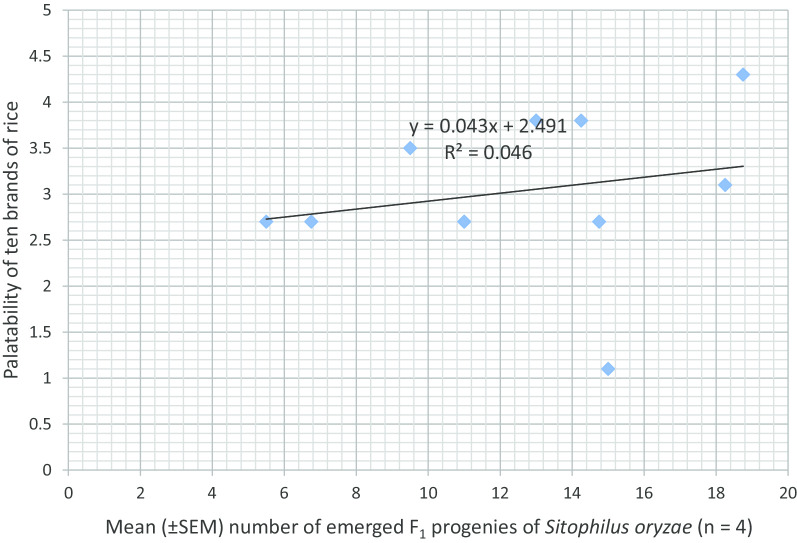
Association between rice brand palatability and number of F_1_ progenies of *Sitophilus oryzae*

**Fig. 2:**
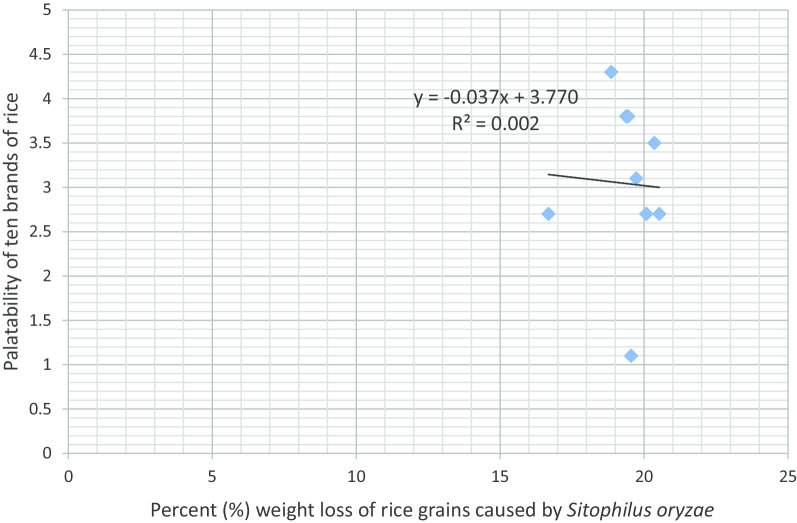
Association between rice brand palatability and grain weight loss caused by *Sitophilus oryzae*

**Fig. 3:**
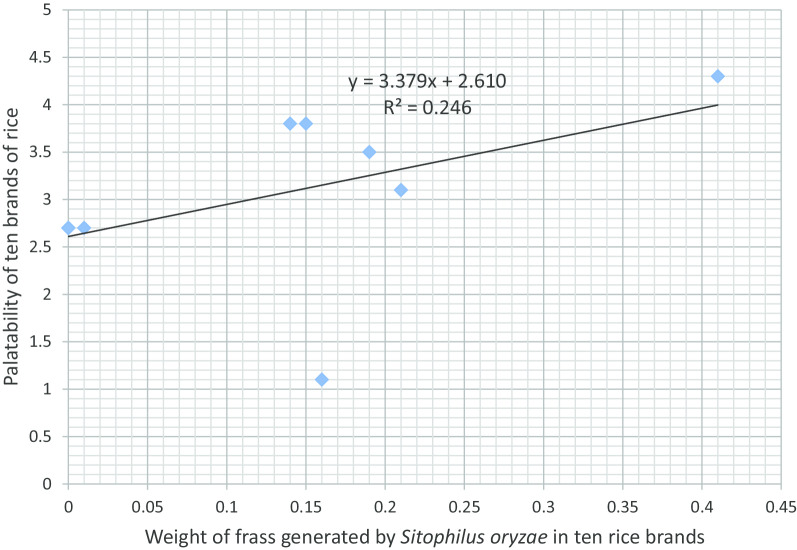
Association between rice brand palatability and weight of frass generated by *Sitophilus oryzae*

**Fig. 4:**
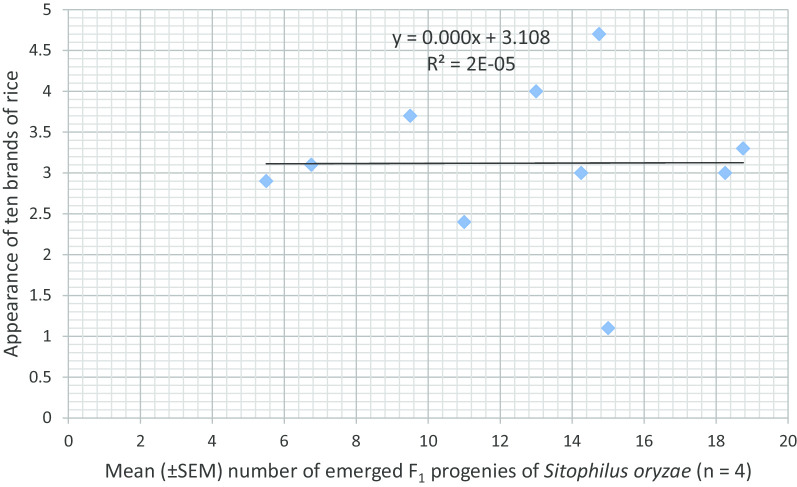
Association between rice brand appearance and number of F_1_ progenies of *Sitophilus oryzae*

**Fig. 5:**
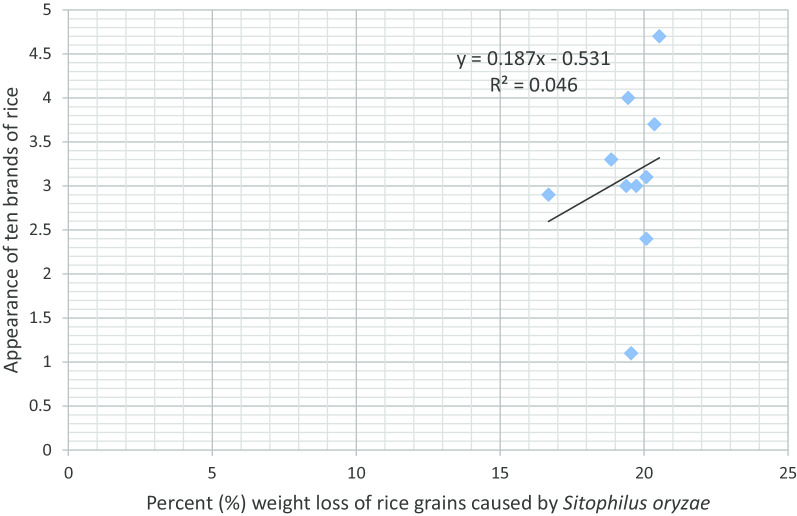
Association between rice brand appearance and grain weight loss caused by *Sitophilus oryzae*

**Fig. 6:**
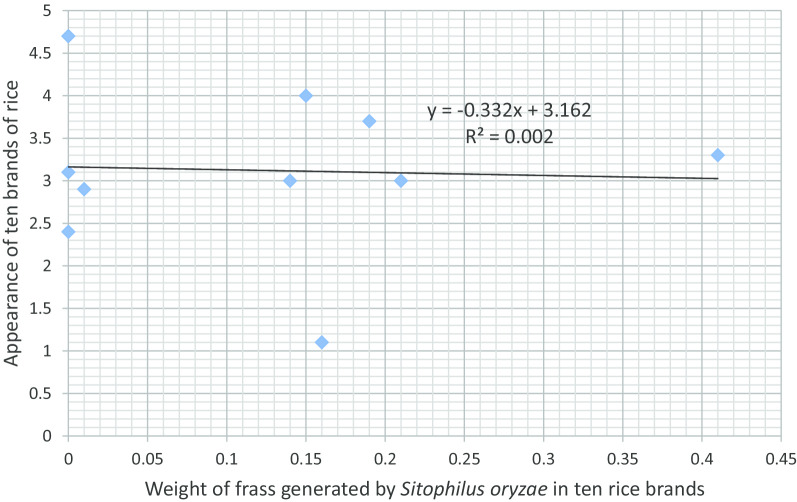
Association between rice brand appearance and weight of frass generated by *Sitophilus oryzae*

Figure 1 presents the association between rice brand palatability and number of F_1_ progenies of *S*. *oryzae*. An increase in the palatability of rice increased the number of F_1_ progenies of *S. oryzae*. Figure 2 shows the association between rice brand palatability and grain weight loss caused by the weevil. An increase in the palatability of rice reduced percent weight loss caused by *S*. *oryzae*. The association between rice brand palatability and weight of frass is presented in Fig. 3. An increase in the palatability of rice increased the weight of frass in the different rice brands. Figure 4 shows the association between appearance of rice brand and number of F_1_ progenies of *S*. *oryzae*. Appearance of rice brand was not related to proliferation of progenies. The association between rice brand appearance and grain weight loss caused by *S*. *oryzae* is shown in Fig. 5. Good appearance of rice grains increased grain weight loss due to insect attack. Figure 6 shows the association between rice brand appearance and weight of frass associated with *S*. *oryzae* activities. Good appearance of rice reduced the quantity of frass generated by *S*. *oryzae*.

## Discussion

This study revealed the extent of economic importance of the rice weevil to rice grains of different brands (local and exotic) stored for 35 days unprotected. The rice weevil, *S*. *oryzae*, has been reported as the commonest coleopteran insect pests of stored white and brown rice (Kamara et al. 2014). As a result, several workers have examined the activities of *S*. *oryzae* on stored rice recording varying levels of economic importance as a consequence of infestation (Ashamo 2005; Kamara et al. 2014; January et al. 2020). The emergence of rice weevil adults, grain weight loss and presence of frass in all the rice brands investigated shows that none of the brands is resistant to infestation and damage by *S*. *oryzae*. Our results indicated that the degree of susceptibility varied from one brand to other, and this is not surprising because different brands may come from different genotypes and even when different brands are produced from same genotype, quality of processing may impart susceptibility differences. The assertion on influence of processing quality is supported by Trematerra et al. (1999).

Although the exotic rice brands came from improved grain post-harvest systems with better processing and handling, they did not perform better than the local rice brand (Abakiliki) in terms of resistance to *S*. *oryzae*. Our findings are not consistent with the belief that local rice products are more susceptible to *S*. *oryzae* than imported products. Similar results were obtained by Khan and Halder (2012). This suggests that rice brand resistance to weevils is not entirely dependent on the efficiency of the post-harvest systems but could also be attributed to origin and other genetic factors. A particular variety can be designated by different brand names and marketed by different companies. McGaughey (1974), Trematerra et al. (1999) and Kamara et al. (2014) reported that variety that passes through more sophisticated operating systems and experiences better processing and handling would likely result to a brand with highly polished grains. They also added that these factors affect grain susceptibility to insect attack.

*Sitophilus oryzae* successfully fed and developed in the grains of the sampled rice brands, showing that rice (source of starch and protein) is not only required in human nutrition but also needed by insects as staple. This is not surprising because it has been reported that weevils require starch and protein in order to grow and to lay eggs (Nwosu 2016). Rice contains 70 to 80% starch, 7% protein, 1.5% oils and vitamins A, B and C (Kochhar 1986). Thus weevils feed on rice grains to obtain also oils and vitamins for their daily metabolism. Weight loss suffered by the various rice brands is strongly attributed to the biological activities of the storage insect pest. Although there were variations in grain weight loss among the different rice brands, an average of 19.47% weight loss was revealed. This appears to be lower than weight loss recorded for other staples in the traditional storage system and in consistent with our field experience; stored rice suffers lower destruction by storage insect pests than maize and legumes. In India, grain weight loss due to the attack of storage insects has been estimated at 5 to 25% (Prakash and Rao 1995), and this is similar to the weight loss recorded in the present study. Frass accumulation in each rice brand at the end of storage is also strongly attributed to the biological activities of the rice weevil. This corroborates with the findings of Ofuya and Lale (2001) that feeding and developmental activities of grubs culminate in extensive economic injury to infested grains and thus leaving the grains hollow, and damage ultimately manifest in severe powdering of the grains.

Analyses of results on *S*. *oryzae* body weight, rice palatability and appearance and their relationships with infestation rate revealed useful food quality and safety information. Though the degree of rice susceptibility to rice weevil pest differed from one brand to the other, earlier workers have associated susceptibility to grain hardness, other physical properties and some chemical characteristics (Ahmad et al. 1998; Astuti et al. 2013). In the present study, we have found rice palatability and appearance important in *S*. *oryzae* infestation of rice, irrespective of origin, i.e., whether foreign or local. The results revealed that palatable rice supported the emergence of new F_1_ progenies of *S*. *oryzae* and inadvertently allowed high accumulation of frass in rice which accelerates damage and rejection. Our findings further revealed that grain appearance was not related to progeny emergence; thus, such good-looking grains did not support production/accumulation of frass in rice. Good appearance reduced grain damage by *S*. *oryzae* and increased visual appeal. Interestingly, good appearance also reduced the weight of male and female insects at emergence. This information is important for managers seeking to minimize rice weevil infestation in stores.

## Conclusions

The study on the susceptibility of different rice brands to *S*. *oryzae* shows that none was resistant to infestation and damage by the weevil. Males that were bred on the different rice brands did not differ in weight at emergence, but the weight of *S*. *oryzae* females at emergence was influenced by brand difference, suggesting that the female insects are more sensitive to brand difference in terms of weight gains. The present study has revealed the importance of grain palatability and appearance in *S*. *oryzae* infestation of stored rice. The proliferation of diverse genotypes and hybrids of rice (branded) with enhanced resistance to *S*. *oryzae* should be strongly encouraged to reduce susceptibility and increase rice security. Industries should ensure that rice grains have clean/good appearance before branding to discourage frass accumulation and reduce damage. These measures will help greatly to minimize infestation and damage by the rice weevil.

## Data Availability

Data collected and analyzed during the current study are available from the corresponding author on sensible request.
